# Posttranslational Regulation of IL-23 Production Distinguishes the Innate Immune Responses to Live Toxigenic versus Heat-Inactivated Vibrio cholerae

**DOI:** 10.1128/mSphere.00206-19

**Published:** 2019-08-21

**Authors:** Ana A. Weil, Crystal N. Ellis, Meti D. Debela, Taufiqur R. Bhuiyan, Rasheduzzaman Rashu, Daniel L. Bourque, Ashraful I. Khan, Fahima Chowdhury, Regina C. LaRocque, Richelle C. Charles, Edward T. Ryan, Stephen B. Calderwood, Firdausi Qadri, Jason B. Harris

**Affiliations:** a Infectious Diseases Division, Massachusetts General Hospital, Boston, Massachusetts, USA; b Department of Medicine, Harvard Medical School, Boston, Massachusetts, USA; c Infectious Diseases Division, International Center for Diarrheal Disease and Research, Bangladesh (icddr,b), Dhaka, Bangladesh; d Department of Immunology and Infectious Diseases, Harvard T. H. Chan School of Public Health, Boston, Massachusetts, USA; e Department of Microbiology, Harvard Medical School, Boston, Massachusetts, USA; f Department of Pediatrics, Harvard Medical School, Boston, Massachusetts, USA; g Division of Global Health, Massachusetts General Hospital for Children, Boston, Massachusetts, USA; University of Maryland School of Medicine

**Keywords:** IL-23, *Vibrio cholerae*, cholera

## Abstract

Vibrio cholerae infection provides long-lasting protective immunity, while oral, inactivated cholera vaccines (OCV) result in more-limited protection. To identify characteristics of the innate immune response that may distinguish natural V. cholerae infection from OCV, we stimulated differentiated, macrophage-like THP-1 cells with live versus heat-inactivated V. cholerae with and without endogenous or exogenous cholera holotoxin (CT). Interleukin 23A gene (*IL23A*) expression was higher in cells exposed to live V. cholerae than in cells exposed to inactivated organisms (mean change, 38-fold; 95% confidence interval [95% CI], 4.0 to 42; *P* < 0.01). IL-23 secretion was also higher in cells exposed to live V. cholerae than in cells exposed to inactivated V. cholerae (mean change, 5.6-fold; 95% CI, 4.4 to 11; *P* < 0.001). This increase in IL-23 secretion was more marked than for other key innate immune cytokines (e.g., IL-1β and IL-6) and dependent on exposure to the combination of both live V. cholerae and CT. While IL-23 secretion was reduced following stimulation with either heat-inactivated wild-type V. cholerae or a live isogenic *ctxAB* mutant of V. cholerae, the addition of exogenous CT restored IL-23 secretion in combination with the live isogenic *ctxAB* mutant V. cholerae, but not when it was paired with stimulation by heat-inactivated V. cholerae. The posttranslational regulation of IL-23 under these conditions was dependent on the activity of the cysteine protease cathepsin B. In humans, IL-23 promotes the differentiation of Th17 cells to T follicular helper cells, which maintain and support long-term memory B cell generation after infection. Based on these findings, the stimulation of IL-23 production may be a determinant of protective immunity following V. cholerae infection.

**IMPORTANCE** An episode of cholera provides better protection against reinfection than oral cholera vaccines, and the reasons for this are still under study. To better understand this, we compared the immune responses of human cells exposed to live Vibrio cholerae with those of cells exposed to heat-killed V. cholerae (similar to the contents of oral cholera vaccines). We also compared the effects of active cholera toxin and the inactive cholera toxin B subunit (which is included in some cholera vaccines). One key immune signaling molecule, IL-23, was uniquely produced in response to the combination of live bacteria and active cholera holotoxin. Stimulation with V. cholerae that did not produce the active toxin or was killed did not produce an IL-23 response. The stimulation of IL-23 production by cholera toxin-producing V. cholerae may be important in conferring long-term immunity after cholera.

## Abstract (precis)

An episode of cholera provides better protection against reinfection than oral cholera vaccines, and the reasons for this are still under study. To better understand this, we compared the immune responses of human cells exposed to live Vibrio cholerae with those of cells exposed to heat-killed V. cholerae (similar to the contents of oral cholera vaccines). We also compared the effects of active cholera toxin and the inactive cholera toxin B subunit (which is included in some cholera vaccines). One key immune signaling molecule, IL-23, was uniquely produced in response to the combination of live bacteria and active cholera holotoxin. Stimulation with V. cholerae that did not produce the active toxin or was killed did not produce an IL-23 response. The stimulation of IL-23 production by cholera toxin-producing V. cholerae may be important in conferring long-term immunity after cholera.

## INTRODUCTION

Vibrio cholerae causes cholera, a severe acute secretory diarrhea. There are an estimated 3 million cases of cholera and 100,000 deaths annually, with at least half of the deaths occurring in children under 5 years (1, 2). Only two serogroups of V. cholerae, O1 and O139, have caused widespread cholera. V. cholerae O1 biotype El Tor is the cause of the current global cholera pandemic, while the classical (biotype) V. cholerae O1 caused previous pandemics. V. cholerae O1 is further classified into two main serotypes, Inaba and Ogawa, which differ by the presence of a methyl group on the lipopolysaccharide (LPS) in strains of the Ogawa serotype.

Vaccination is an essential tool for reducing the burden of cholera, and three major types of oral cholera vaccines (OCV) are currently licensed and commercially available. An attenuated vaccine, CVD 103-HgR (Vaxchora, Paxvax), was engineered from a classical V. cholerae O1 strain and expresses the inactive cholera toxin B subunit (CTB). It is currently recommended for U.S. travelers at high risk of exposure. An inactivated vaccine, WC-rBS (Dukoral, Valneva), contains a mixture of heat- and formalin-inactivated classical and El Tor V. cholerae O1 and includes the recombinant CTB. This vaccine is used primarily by travelers from Europe and Canada. A bivalent inactivated whole-cell vaccine (WC vaccine; produced and marketed either as Shanchol [Shantha Biotechnics] or Euvichol [EuBiologics]) includes both heat- and formalin-inactivated strains of the El Tor O1 Ogawa and Inaba and O139 serogroups but does not include CTB. From a public health standpoint, the WC vaccine is the most widely used globally, both in areas of cholera endemicity and to respond to and prevent cholera epidemics and humanitarian crises.

Studies of immune responses to natural infection compared to responses to OCV suggest that immunity elicited by natural infection may be longer lasting and is quantitatively different, especially in children. Natural infection and OCV stimulate specific gut-homing and T follicular helper (Tfh) cell responses that are present in adults but diminished in older children that receive WC-rBS, and these responses are nearly absent in children under 5 years (3–5). Immune responses to V. cholerae antigens are believed to contribute to long-term protection, and immune responses are significantly reduced in young children after receiving WC-rBS or the WC vaccine compared to responses in adults (6, 7). While the WC OCV seems to confer durable protection in older children and adults for at least 3 to 5 years, protection remains suboptimal in younger children, especially those under 2 years of age (8, 9). The mechanistic and kinetic differences in immunologic stimulation after natural infection and vaccination and how these responses relate to long-term protection are not well understood (10, 11).

A series of *in vitro*, genetic, and genomic studies indicate that a broad innate immune response occurs after cholera infections (12–14). Genome-wide association studies of persons from areas of cholera endemicity have demonstrated strong selection for genes required for the activation of nuclear factor kappa light-chain enhancer of activated B cells (NF-κB), a major regulator of transcriptional genes that elicit innate immune responses to bacterial pathogens (15). V. cholerae colonizes in the small intestine, and in our previous studies of secreted proteins from biopsy specimens of human duodenal mucosa during cholera, we found that major modulators of innate immune responses, including Toll-like receptor 4 (TLR4), NF-κB, and caspase-dependent inflammasomes, were differentially abundant during acute disease (16). These data were further supported by a study of gene expression in duodenal biopsy specimens from cholera patients from whom acute- and convalescent-phase samples were compared; NF-κB signaling was found to be the most upregulated pathway in acute disease (17), and IL-1β, IL-6, and IL-23 were identified as top upstream regulators during acute infection (16, 17).

In the context of the increasing evidence for the role of innate immune responses in V. cholerae protective immunity and observed differences in natural and OCV innate immune responses, we sought to identify and characterize these differences using a cell culture model of V. cholerae infection. We differentiated human THP-1 monocytes into macrophage-like cells, a system that has been used previously for testing innate immune responses to vaccine antigens in comparison to responses to natural infection (17–19). We stimulated cells with live and heat-inactivated V. cholerae with and without endogenous or exogenous CT to model differences between innate immune responses to natural V. cholerae infection and responses to OCV. Gene expression differences under these conditions suggested that IL-23 was produced most abundantly after cells were stimulated with live V. cholerae and CT. The secretion of this cytokine was posttranscriptionally regulated and required the activity of the protease cathepsin B for secretion.

## RESULTS

### *IL23A* expression is upregulated by exposure of cells to live toxigenic V. cholerae O1 relative to levels of expression after exposure to heat-inactivated organisms on a transcriptome-wide screen.

**FIG 1:**
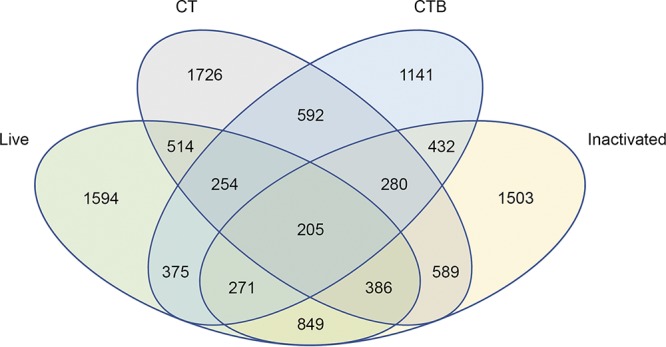
Gene expression under various stimulation conditions and overlap between expression profiles. RNA-seq was performed on PMA-induced THP-1 cells after stimulation with live V. cholerae (Live), heat-inactivated V. cholerae (Inactivated), recombinant cholera toxin (CT), and cholera toxin subunit B (CTB). Genes for which the log_2_ number of fragments per kilobase per million (FPKM) divided by the number of FPKM without stimulation (medium value) was >0.99 are listed. Genes with a zero value for the number of FPKM for either stimulated or unstimulated wells were excluded.

To begin modeling differences in innate immune responses between vaccination and natural infection, we compared levels of RNA expression from differentiated THP-1 cells stimulated with either live or heat-inactivated V. cholerae (grown under toxin-inducing conditions) in cells stimulated with active CT or CTB. RNA integrity numbers ranged from 8.9 to 9.6 (median, 9.1), with a sequencing depth between 12 and 48 million reads per sample (median, 18.9 million). No samples were excluded based on RNA quality. Expression profiles were benchmarked against an unstimulated control (medium only), and genes differentially expressed in response to a stimulus were identified by log_2_ ratio analysis. The total genes expressed numbered 23,285. The breakdown for and overlap of genes without zero values for stimulated or unstimulated wells and with fragments per kilobase per million (FPKM) values for stimulated over unstimulated (medium) log_2_ values of >0.99 are shown in Fig. 1. We found that *IL23A* expression was 29-fold higher in samples stimulated by exposure to live bacteria than in samples stimulated by exposure to inactivated bacteria.

### Exposure to live V. cholerae and cholera holotoxin are required for maximal IL-23 secretion.

Because the transcriptome-wide analysis of THP-1 responses suggested that *IL23A* expression was strongly differentially regulated by exposure to live versus heat-inactivated V. cholerae and because our previous studies from human duodenal tissue samples provided evidence that IL-23 is an important mediator of the innate immune response to acute cholera (17), we conducted further experiments to elucidate the dynamics of IL-23 expression and secretion using our model. In these subsequent experiments, we assessed the generation of both mRNA and protein secretion. We measured *IL23A* gene expression in differentiated THP-1 human monocytes by quantitative real-time PCR (qRT-PCR) and IL-23 secretion into the cell supernatant using an enzyme-linked immunosorbent assay (ELISA). We tested 17 experimental replicates of the stimulated conditions and excluded four experiments. Three were excluded based on inadequate stimulation in the positive-control wells (defined as an average of <1,000 pg/ml of IL-23 secretion), and a fourth experiment was excluded due to a high level of variation between the three negative- and positive-control wells (see Materials and Methods).

**FIG 2:**
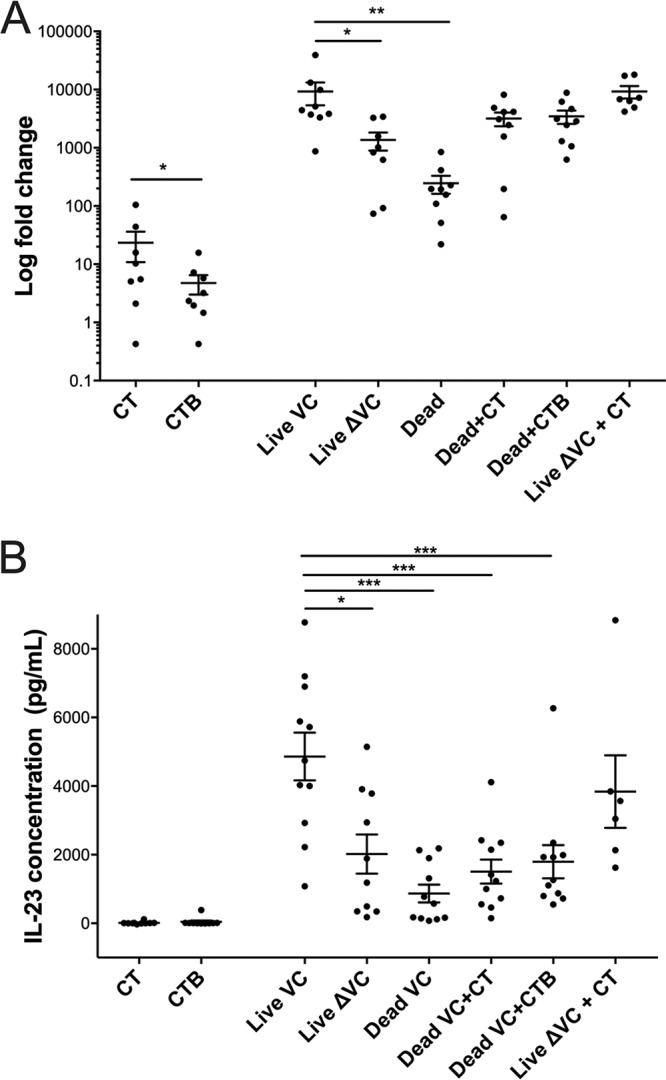
*IL23* is expressed and secreted in response to stimulation of differentiated THP-1 cells with live toxigenic V. cholerae O1. *IL23* expression (A) and secretion (B) were measured in response to recombinant CT, CTB, live V. cholerae N16961 (Live), live V. cholerae Δ*ctxAB* JBK70 (Live ΔVC), heat-inactivated wild-type V. cholerae N16961 (Inactivated), heat-inactivated wild-type V. cholerae N16961 with CT (Inactivated VC + CT), heat-inactivated wild-type V. cholerae N16961 with CTB (Inactivated VC + CTB), and live V. cholerae Δ*ctxAB* JBK70 with CT (Live ΔVC + CT). Each point represents the mean of results from three technical replicates from a single experiment, while the horizontal bars represent mean values across multiple biological replications of the cell culture stimulation experiment. Error bars represent standard errors of the means. Expression was determined by qRT-PCR, and IL-23 secretion was determined by ELISA using cells/supernatants collected after 24 h of stimulation. ***, *P* < 0.001; **, *P* < 0.01; *, *P* < 0.05 (by paired nonparametric testing).

As shown in Fig. 2, CT and CTB alone induced limited *IL23* expression (Fig. 2A) and no detectable secretion of IL-23 in the cultured cell supernatants (Fig. 2B). Expression of *IL23* was much higher in response to exposure to live V. cholerae than in response to inactivated bacteria (mean change, 38-fold; 95% CI, 4.0 to 42; *P* < 0.01) and higher in live wild-type toxin-producing V. cholerae than in otherwise-isogenic V. cholerae *ΔctxAB*. However, the addition of CT or CTB to heat-inactivated V. cholerae increased *IL23A* expression to the point that there was no longer a significant difference in the levels of expression between cells stimulated with live and cells stimulated with heat-inactivated V. cholerae with added exogenous holotoxin.

The secretion of IL-23 was also dependent on the combination of live V. cholerae and CT. Compared to stimulation with both heat-inactivated V. cholerae and isogenic live V. cholerae *ΔctxAB*, stimulation with a live holotoxin-producing strain resulted in significantly more IL-23 secretion (live holotoxin-producing strain compared to heat-inactivated V. cholerae, mean change, 5.6-fold; 95% CI, 4.4 to 11; live holotoxin-producing strain compared to isogenic live V. cholerae *ΔctxAB*, mean change, 2.4-fold; 95% CI, 1.9 to 4.5 [for both, *P* was <0.001]). However, unlike with expression, there was no increase in secreted IL-23 observed after stimulation with heat-inactivated V. cholerae following the addition of CT or CTB to the heat-inactivated bacteria. In contrast, IL-23 secretion was restored to the level generated by stimulation with wild-type V. cholerae by the addition of exogenous CT to the isogenic V. cholerae *ΔctxAB* mutant.

### IL-1β and IL-6 demonstrated variable regulation after infection with live compared to heat-inactivated V. cholerae.

**FIG 3:**
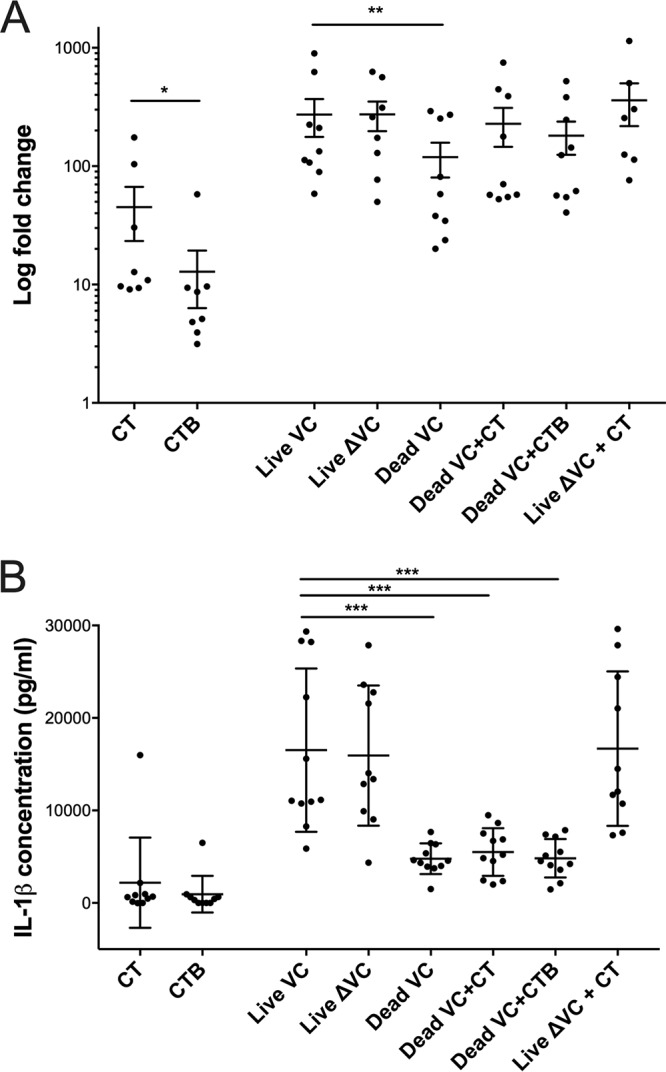
*IL1β* expression and secretion in response to stimulation of differentiated THP-1 cells with live toxigenic V. cholerae O1. *IL1β* expression (A) and secretion (B) were measured in response to stimulation with CT, CTB, live V. cholerae N16961 (Live VC), live V. cholerae Δ*ctxAB* JBK70 (Live ΔVC), inactivated V. cholerae N16961 (Inactivated VC), inactivated V. cholerae N16961 plus CT (Inactivated VC + CT), inactivated V. cholerae N16961 plus CTB (Inactivated VC + CTB), and live Δ*ctxAB* JBK70 (Live ΔVC + CT). Horizontal bars represent mean values across multiple biological replications of the cell culture stimulation experiment, and error bars represent standard errors of the means. Expression was determined by qRT-PCR analysis of the *IL1β* gene using cDNA from THP-1 cells after 24 h of stimulation. Expression levels were quantified relative to levels in an unstimulated control (medium) and the housekeeping gene *ACTB* (β actin) using the ΔΔ*C_T_* method of qRT-PCR analysis, expressed as fold change on a logarithmic scale. IL-1β secretion from THP-1 cells was determined by ELISA using supernatants collected after 24 h of stimulation. IL-1β protein concentration is represented in picograms of the supernatant per milliliter. ***, *P* < 0.001; **, *P* < 0.01; *, *P* < 0.05 (by paired nonparametric testing).

We measured the expression and secretion of IL-1β, a cytokine whose secretion is dependent on the activation of the NLRP3 inflammasome, a pathway shared by IL-23 (15–17, 20). The activation of the NLRP3 inflammasome results in the cleavage of caspase-1, which in turn cleaves the inactive form of cytokine precursors into the active form (21). Some features of IL-1β responses were similar to IL-23 responses, including IL-1β expression and secretion increases in response to live V. cholerae compared to levels in response to heat-inactivated bacteria (mean change, 2.3-fold; 95% CI, 1.7 to 2.4; and mean change, 3.5-fold; 95% CI, 2.9 to 3.8 [for both, *P* was <0.001], respectively) (Fig. 3). CT stimulation resulted in more *IL1β* expression than CTB stimulation (mean change, 2.3-fold; 95% CI 1.7 to 2.4; *P* < 0.05). Live V. cholerae or heat-inactivated bacteria plus CT were also effective in stimulating IL-1β expression. However, unlike with IL-23 secretion, there was no difference in the levels of IL-1β secretion between wild-type and otherwise-isogenic nontoxigenic V. cholerae, and the addition of CT to live V. cholerae *ΔctxAB* did not increase IL-1β secretion.

**FIG 4:**
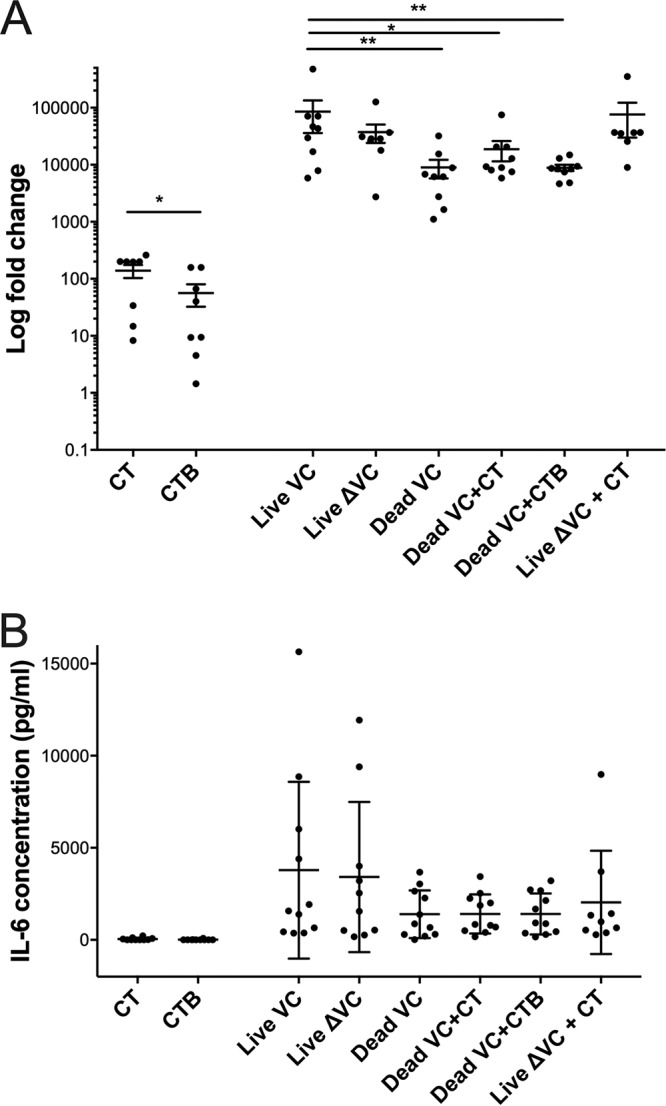
*IL6* expression and secretion in response to stimulation of differentiated THP-1 cells with live toxigenic V. cholerae O1. *IL6* expression (A) and secretion (B) in response to stimulation with CT, CTB, live V. cholerae N16961 (Live VC), live V. cholerae Δ*ctxAB* JBK70 (Live ΔVC), inactivated V. cholerae N16961 (Inactivated VC), inactivated V. cholerae N16961 plus CT (Inactivated VC+CT), inactivated V. cholerae N16961 plus CTB (Inactivated VC+CTB), and live V. cholerae Δ*ctxAB* JBK70 plus CT (Live ΔVC + CT). Horizontal bars represent mean values across multiple biological replications of the cell culture stimulation experiment, and error bars represent standard errors of the means. Expression was determined by qRT-PCR analysis of the *IL6* gene using cDNA from THP-1 cells after 24 h of stimulation. Expression levels were quantified relative to those of an unstimulated control (medium) and the *ACTB* housekeeping gene (β-actin) using the ΔΔ*C_T_* method of qRT-PCR analysis, expressed as fold change on a logarithmic scale. IL-6 secretion from THP-1 cells was determined by ELISA using supernatants collected after 24 h of stimulation. IL-6 protein concentration is represented in picograms of the supernatant per milliliter. **, *P* < 0.01; *, *P* < 0.05 (by paired nonparametric testing).

To distinguish additional innate immune pathways differentially activated by exposure to live compared to heat-inactivated V. cholerae, we next measured responses to IL-6, another proinflammatory cytokine that was previously found to be upregulated during acute cholera (16, 17). *IL6* expression from macrophages is dependent on NF-κB activation and triggered by pathogen-associated molecular patterns via TLR4 activation, also in an NLRP3-independent pathway (16, 22, 23). We found that *IL6* expression was highest in response to live bacteria compared to inactivated bacteria, similar to the pattern seen with *IL23* and *IL1β* (mean change, 9.5-fold; 95% CI, −18 to 12; *P* < 0.01) (Fig. 4). Although *IL6* expression in response to holotoxin alone was much lower than its expression when bacteria were present, CT stimulated more expression than CTB (mean change, 2.5-fold; 95% CI, 2 to 640; *P* < 0.05). However, unlike with IL-23 and IL-1β, we found no difference in IL-6 secretion between stimulation with live bacteria and stimulation with heat-inactivated bacteria, and IL-6 production was not dependent upon live V. cholerae (live compared to heat-inactivated stimulation mean change, 2.6-fold; 95% CI, 1.1 to 3.1; *P* = 0.20). The addition of cholera holotoxin did not alter the secretion of IL-6 after exposure to live V. cholerae compared to that after exposure to live V. cholerae *ΔctxAB* with CT (mean change, 1.8-fold; 95% CI, −4.6 to 1.7; *P* = 0.50).

### IL-23 secretion is posttranscriptionally regulated by the lysosomal enzyme cathepsin B.

**FIG 5:**
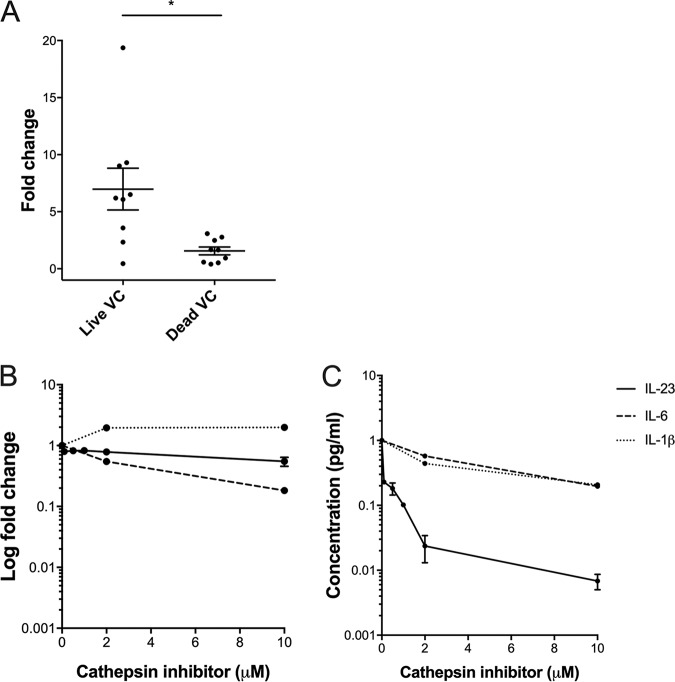
Induction of inflammatory cytokines in response to stimulation of human THP-1 cells with wild-type V. cholerae in the presence of a cathepsin B inhibitor. (A) Cathepsin B expression (expressed as fold change) in THP-1 monocytes in response to live V. cholerae N16961 (Live VC) or heat-inactivated V. cholerae N16961 (Inactivated VC). Horizontal bars represent mean values across multiple biological replications of the cell culture stimulation experiment, and error bars represent standard errors of the means. *, *P* < 0.05 (by paired nonparametric testing). *IL23A*, *IL6*, and *IL1β* expression (B) and secretion (C) in THP-1 cells incubated with various concentrations of the cathepsin B inhibitor for 3 h and then exposed to live or heat-inactivated V. cholerae N16961 for 1 h. Expression was determined by qRT-PCR analysis of the *IL23A*, *IL6*, and *IL1β* genes, respectively, using cDNA from THP-1 cells. Expression levels were quantified relative to expression in an unstimulated control (medium) and expression of the housekeeping gene *ACTB* (β actin) using the ΔΔ*C_T_* method of qRT-PCR analysis. Expression data are represented on a logarithmic scale (log_2_ ΔΔ*C_T_*) , expressed as fold change. IL-23, IL-6, and IL-1β secretion from THP-1 cells was determined by ELISA using the THP-1 cell supernatant. Experiments were conducted in replicates of two, and error bars represent standard errors of the means.

IL-1β and IL-23 regulation are at least partially controlled by the enzyme caspase-1, which is in turn regulated by inflammasome activation in human macrophages and monocytes (24). Caspase-1 cleaves the procytokine IL-1β into its active form, and the mechanism of caspase-1 activation of IL-23 production is presumed to also be through cleavage, the primary function of caspase-1 (20). IL-23 secretion has also been found to be dependent on cathepsin B, a lysosomal protease, which is thought to exit the lysosome and act upstream of the NLRP3 inflammasome to regulate caspase-1 (20, 25). In order to define the activation pathways discriminating between maximal IL-1β and IL-23 production, we measured cathepsin B expression levels in THP-1 cells stimulated with live and inactivated V. cholerae and found that live bacteria stimulated more cathepsin B production (mean change, 4.5-fold; 95% CI, 3.6 to 4.8; *P* = 0.01) (Fig. 5A). In addition, while *IL23* expression was not affected by the addition of a cathepsin B inhibitor, IL-23 secretion was reduced by a cathepsin B inhibitor in a dose-dependent manner. In contrast, we found that IL-1β and IL-6 secretion were not affected by the addition of a cathepsin B inhibitor. This finding supports the posttranscriptional regulation of IL-23 secretion by cathepsin B as a potential mechanism for the above findings (Fig. 5B and C).

### Components of V. cholerae toxin and simulation of toxin activity induce IL-23 production at a lower magnitude than with live bacteria.

**FIG 6:**
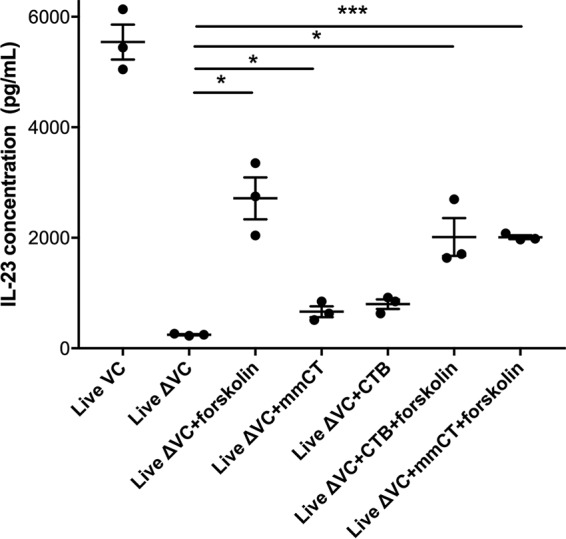
IL-23 secretion from differentiated THP-1 cells after stimulation with live V. cholerae is partially recapitulated using forskolin, which induces cAMP activity. IL-23 secretion was measured in response to live V. cholerae N16961 (Live), live V. cholerae Δ*ctxAB* JBK70 (Live ΔVC), live V. cholerae Δ*ctxAB* JBK70 with forskolin (Live ΔVC+forskolin), live V. cholerae Δ*ctxAB* JBK70 with multiple-mutation cholera toxin (Live ΔVC+mmCT), live V. cholerae Δ*ctxAB* JBK70 with cholera toxin B (Live ΔVC+CTB), live V. cholerae Δ*ctxAB* JBK70 with cholera toxin B and forskolin (Live ΔVC+CTB+forskolin), and live V. cholerae Δ*ctxAB* JBK70 with mmCT and forskolin (Live ΔVC+mmCT+forskolin). Each point represents the mean of results from three technical replicates from a single experiment, while the horizontal bars represent mean values across multiple biological replications of the cell culture stimulation experiment. Error bars represent standard errors of the means. IL-23 secretion was determined by ELISA using the number of cells per supernatant collected after 24 h of stimulation. ***, *P* < 0.001; *, *P* < 0.05 (by paired testing [parametric due to the small sample size]).

After observing that both live bacteria and holotoxin were needed for maximal IL-23 secretion, we used additional stimulants to identify more closely the mechanism or components of bacteria necessary for the greatest IL-23 secretion. We added a mixture of bacterial proteins derived from the membrane of V. cholerae N16961 (MP) to differentiated THP-1 cells (26) and found that IL-23 secretion did not approach that measured after stimulation with live bacteria (mean increase in IL-23 from cells stimulated with live bacteria compared to those stimulated with MP, 3.4-fold; 95% CI, −1.1 to 1.1; *P* < 0.05) (see Fig. S1 in the supplemental material). We then used forskolin to reproduce the enzymatic activity of cholera toxin A by activating adenylyl cyclase to increase intracellular levels of cyclic AMP (cAMP). The combination of forskolin and live V. cholerae *ΔctxAB* resulted in an increase in IL-23 secretion compared to levels after infection with live V. cholerae *ΔctxAB* alone (mean change, 11-fold; 95% CI, 5.3 to 15; *P* < 0.05), although this did not approach the level of IL-23 produced by live bacteria (Fig. 6). We also included an inactive cholera toxin with mutations in both CTA and CTB (multiple-mutation CT [mmCT]), and when it was combined with the live V. cholerae *ΔctxAB*, we observed a modest increase in IL-23 secretion from stimulated cells compared to the level secreted with live V. cholerae *ΔctxAB* alone (mean change, 2.7-fold; 95% CI, 1.2 to 8.8; *P* < 0.05). The IL-23 secretion from cells stimulated with mmCT plus live V. cholerae *ΔctxAB* was not significantly different than that after the stimulation provided by CTB with live V. cholerae *ΔctxAB* (Fig. 6). The addition of mmCT to forskolin with live V. cholerae *ΔctxAB* did not result in IL-23 secretion beyond the stimulation provided by only forskolin with live V. cholerae *ΔctxAB* and still did not approach the IL-23 secretion observed after the stimulation of cells with live V. cholerae. The experiments including MP stimulation were conducted at an earlier time point and therefore could not be combined with the forskolin and mmCT trials.

10.1128/mSphere.00206-19.1 FIG S1 IL-23 secretion from differentiated THP-1 cells after stimulation with V. cholerae membrane preparation is low. IL-23 secretion was measured in response to live V. cholerae N16961 (Live), live V. cholerae Δ*ctxAB* JBK70 (Live ΔVC), heat-inactivated wild-type V. cholerae N16961 (Inactivated), inactivated V. cholerae with cholera toxin (Inactivated VC+CT), inactivated VC with cholera toxin B (Live ΔVC+CTB), and a V. cholerae N16961 membrane preparation of outer membrane proteins (MP). Data for Live VC, Live ΔVC, Inactivated VC, Inactivated VC+CT, and Inactivated VC+CTB are also shown in Fig. 2, with accompanying paired-analysis statistical testing. Each point represents the mean of results from three technical replicates from a single experiment, while the horizontal bars represent mean values across multiple biological replications of the cell culture stimulation experiment. Error bars represent standard errors of the means. IL-23 secretion was determined by ELISA using the numbers of cells per supernatant collected after 24 h of stimulation. *, *P* < 0.05 (by unpaired nonparametric testing). Download FIG S1, TIF file, 0.1 MB. Copyright © 2019 Weil et al. 2019 Weil et al. This content is distributed under the terms of the Creative Commons Attribution 4.0 International license.

## DISCUSSION

V. cholerae infection generates immune responses that protect humans from recurrent infection for at least several years (27, 28), while immune responses to inactivated oral cholera vaccines provide more-limited protection than natural infection, particularly in young children (3, 29). This difference in adaptive immunity following infection versus vaccination may originate from a divergence in the innate immune responses to live V. cholerae and to inactivated vaccine composition. To model this *in vitro*, we compared responses of stimulated cultured human monocytes by exposing them to live and heat-inactivated V. cholerae and toxin components. The key novel finding from this study was that IL-23 was highly upregulated in response to exposure of cells to live V. cholerae at the transcriptional and posttranscriptional levels and that posttranscriptional regulation of IL-23 secretion was dependent on stimulation with both live V. cholerae organisms and cholera holotoxin. This *in vitro* finding was specific to IL-23 and did not appear to be broadly applicable to other innate immune cytokine responses, including IL-6 and IL-1β. However, our finding is consistent with those of previous *in vivo* studies that predicted that IL-23 is a key regulator of the innate immune response observed in human duodenal tissue during acute cholera (17).

These *in vitro* findings are also consistent with, and may explain, the divergence in the types of adaptive immune responses following live infection and vaccination in humans. IL-23 is a member of a group of cytokines that stimulate naive T cells to differentiate into Th17 cells, and among this group of cytokines, IL-23 is known to further differentiate Th17 cells into T follicular helper (Tfh) cells (30). Antigen-specific Tfh cells are thought to be critical for the development of protective memory B cell responses that mediate long-term immunity, and a recent study found that circulating Tfh cells in patients with cholera affect subsequent antigen-specific B cell development and consequent immunoglobulin production (31). IL-23 stimulates Th17 cells, and we previously observed that Th17-associated cytokines are activated in cholera patients to a greater degree than in OCV recipients (3). This finding is consistent with our prior findings that the development of Tfh responses after receipt of an OCV correlates with the development of V. cholerae-specific memory B cells (3). Young children, who are not well protected by cholera vaccines compared to adults, have no observed Tfh responses after vaccination (3).

To better understand what drives the activation of IL-23 production after exposure to live V. cholerae, we compared its expression and secretion with those of additional cytokines known to be activated in cholera, including IL-1β and IL-6. Cholera holotoxin is known to activate the NLRP3 inflammasome through several mechanisms that vary by V. cholerae biotype, including dephosphorylation of the pyrin domain, resulting in caspase-1-dependent IL-1β secretion (15, 32–34). The regulations of IL-1β and IL-23 follow similar pathways in that both are transcriptionally activated via the central NF-κB pathway (via LPS and TLR4 activation), and secretion is dependent on activation of the NLRP3 inflammasome, resulting in caspase-1-dependent secretion (20). In our study, we showed that while both *IL1β* and *IL23* were highly expressed in response to exposure of cells to live V. cholerae, maximal IL-1β secretion did not require cholera holotoxin, while maximal IL-23 secretion depended on stimulation with both live organisms and cholera holotoxin. Because previous studies have shown that IL-23 and IL-1β pathways of secretion diverge at the point where cathepsin B regulates IL-23 secretion, we speculated that a mechanism of differential regulation between IL-1β and IL-23 was the action of cathepsin B (20). This was consistent with our data demonstrating that cathepsin B expression was upregulated in response to stimulation with live V. cholerae and that IL-23 secretion required cathepsin B, while IL-1β did not (20, 35). We also measured IL-6 expression and production since IL-6 is dependent on NF-κB activation through an NLRP3-independent pathway (16, 23, 36). We found that IL-6 production was not dependent on exposure to live V. cholerae, consistent with its known activation by NF-κB by LPS alone (37).

We also tested cholera holotoxin (composed of 1 A subunit and 5 B subunits) in comparison to the enzymatically inactive B subunit and to mmCT, a nontoxic inactive holotoxin that has 1,000-fold-reduced cAMP activity compared to that of cholera toxin A (32, 38). These experiments revealed that holotoxin was required for maximal IL-23 secretion. We hypothesize that this difference may be due to the action of cholera toxin subunit A, which is present in natural infection and lacking in OCV recipients. This is supported by our findings that IL-23 secretion increased from cells stimulated with forskolin, which reproduces cholera toxin A enzymatic activity. In the small intestine, the B subunits of cholera toxin bind the ganglioside GM1 at the mucosal surface, resulting in endocytosis of the holotoxin. The endosome then travels to the endoplasmic reticulum, and there a disulfide bond of the A subunit is reduced to generate subunits A1 and A2 (39). In the endoplasmic reticulum, A1 uses a mechanism for retro-translocation of misfolded proteins to escape into the cytosol, where it then is free to activate adenylyl cyclase, inducing diarrhea (25, 33). The mechanism of subunit A1 activation of the endosome is not known. Based on the results reported here, the difference in innate immune responses between naturally infected and vaccinated individuals may be linked to the stimulation provided by exposure to the A1 subunit in the endosome, in addition to the downstream effects of adenylyl cyclase activation.

Our study has limitations. We used immortalized, cultured human-derived monocytes differentiated to macrophages to simulate human infection. We used this model to study differences in immune responses to fixed amounts of bacteria that were live or inactivated, and this served as a proxy for natural infection and vaccination; however, our model lacks many of the physiologic conditions associated with *in vivo* exposure. A critical element of these conditions is likely the effect of signaling and interaction between multiple cell types in the gut mucosa and, potentially, the effect of the gut microbiome influencing host responses to V. cholerae (40). Despite these limitations, we were able to successfully identify differences in stimulation using the specific cell type (macrophages) that regulates lymphocyte responses, which ultimately mediate the development of long-term immunity.

In summary, this report demonstrates that IL-23, a key cytokine in acute immune responses to V. cholerae infection, is differentially regulated at the levels of transcription and secretion *in vitro* in response to live toxigenic V. cholerae and inactivated organisms. We think that this is likely an important divergence in the innate immune response that contributes to subsequent adaptive immunity, as IL-23 is known to promote the development of Th17 into T follicular helper cells that stimulate long-term protective immunity after cholera. Furthermore, our report suggests that active cholera toxin may be required to stimulate the posttranscriptional regulation of IL-23 via cathepsin B. While further work is needed to confirm that this *in vitro* finding is functionally significant, the stimulation of IL-23 production through existing or novel vaccine adjuvants should be explored as a potential mechanism to replicate responses to natural infection and achieve longer-term mucosal immunity through vaccination.

## MATERIALS AND METHODS

### THP-1 differentiation and stimulation.

THP-1 monocytes were added to 24-well tissue culture plates at 10^5^ cells per well in 1 ml Roswell Park Memorial Institute medium supplemented with 20% fetal bovine serum. Suspension monocytes were converted to adherent macrophage-like cells by addition of 50 ng/ml phorbol myristate acetate (PMA; Sigma, St. Louis, MO, USA) and incubation at 37°C in 5% CO_2_ for 48 h. Differentiation of monocytes into macrophage-like cells was confirmed by visual inspection of the cellular morphology, confirmation of adherence, and increased cytoplasmic volume. The differentiated THP-1 cells were stimulated with the addition of 1.5 × 10^7^ CFU/well of live or heat-inactivated bacteria added in isolation or in combination with 1 μg/ml purified CT or CTB (both from List Biologicals, Campbell, CA, USA). V. cholerae strain N16961 and the isogenic Δ*ctxAB* JBK70 strain (a gift from Jim Kaper) were grown under cholera toxin-inducing conditions by inoculating one colony from a streaked LB plate into AKI medium with bicarbonate, grown at 37°C for 6 h without agitation, and then transferred to a flask for overnight growth, with agitation at 37°C. To ensure that live and heat-inactivated samples were equal in numbers of CFU per milliliter, a single overnight culture was used and split into equal volumes for both stimulants, and the number of CFU per milliliter was determined before heat inactivation. CFU-per-milliliter counts were determined after serial dilution, plating onto Luria-Bertani agar, and incubation at 37°C for 24 h; if numbers of CFU per milliliter between samples used for live and heat-inactivated stimulations showed greater than a single log difference, the experiment was not continued. An unstimulated control (medium alone) was included with each experimental run of the model. To prevent cell death and overgrowth of the well with live bacteria and to allow cells time for expression and production after stimulation, a final concentration of 150 μg/ml kanamycin was added 1 h after live or inactivated bacteria were added. Cells were then cultured for a total of 24 h at 37°C in 5% CO_2_. The supernatants were collected, and the cell monolayer was collected by the addition of 0.5 ml TRI Reagent (Sigma, St. Louis, MO, USA). All specimens were frozen immediately at –80°C. The supernatants were used for cytokine ELISAs (IL-6, IL-1β, IL-23), and cell pellets were processed for RNA isolation (whole-transcriptome shotgun sequencing [RNA-seq] and qRT-PCR). For the studies evaluating the effect of cathepsin B inhibition on the cytokine response to the above stimulants, cathepsin B inhibitor II, compound CA074 [*N*-(l-3-trans-propylcarbamoyloxirane-2-carbonyl)-l-isoleucyl-l-proline] (Sigma, St. Louis MO, USA) was used (35). Cathepsin B inhibitor at various concentrations was applied to the PMA-differentiated cells for 3 h at 37°C and 5% CO_2_ before the other stimulants were added. To study the effect of a nontoxic CT on the cytokine response, cells were stimulated with 1 μg/ml ultrapure multiple-mutation cholera toxin, mmCT (gifted from Jan Holmgren) (32, 38). In order to investigate whether cAMP production would induce a cytokine response, 20 μM forskolin (Santa Cruz Biotechnology, Dallas, TX) was used to stimulate the differentiated cells. To differentiate effects of live V. cholerae and V. cholerae membrane proteins alone, we used 20 μl of a membrane preparation of V. cholerae N16961 bacterial proteins as an additional stimulant in an experimental volume of 1 ml (26).

### RNA-seq and differential expression.

RNA-seq was performed on RNA extracted from the cell pellets described above. RNA extractions were performed using TRI Reagent according to the manufacturer’s protocols (Sigma). rRNA was removed using the Illumina Ribo-Zero rRNA kit (Illumina, Inc., San Diego, CA, USA). RNA quality was measured by obtaining RNA integrity numbers using an Agilent Bioanalyzer according to the manufacturer protocols (Agilent Technologies Inc., Palo Alto, CA, USA). We determined that samples with an RNA integrity value of less than 7.9 would be excluded (41). For cDNA library construction, we used the PrepX RNA-seq for Illumina library kit (Clontech, Mountain View, CA, USA) and sequenced the libraries as 100-bp single-end reads on an Illumina HiSeq 2500 high-throughput sequencing system. Reads were aligned to the human reference genome UCSC Hg19 using Tophat2, an alignment program that annotates exons and quantifies RNA reads as fragments per kilobase per million (FPKM). To measure differential expression of genes between the stimulated samples and unstimulated controls, we used Cufflinks/Cuffdiff, a program for transcriptome assembly and differential expression of gene transcripts. Gene expression was expressed as a log_2_ fold change between the value for the stimulated sample (live, inactivated, CT, or CTB) and the medium value (42, 43).

### qRT-PCR.

qRT-PCR was used to measure the expression of cytokines *IL6*, *IL1β*, and *IL23*, as well as the enzyme cathepsin B. RNA was purified as described above, and cDNA libraries were prepared using the high-capacity cDNA reverse transcription kit (Applied Biosystems, Vilnius, Lithuania). Target genes were amplified from 25 ng cDNA using a FastStart universal SYBR green I (Rox; Roche Diagnostics, Indianapolis, IN, USA) master mix (10 μl) and specific primers at a concentration of 3.3 μM in a final volume of 15 μl. Reactions were performed in a MicroAmp Fast 96-well reaction plate (Applied Biosystems, Foster City, CA, USA), and each sample was run using 3 replicates to control for variability of PCR amplification. The β-actin housekeeping gene was used to control for variations in cDNA concentration. The following cycling protocol was run using the Applied Biosystems 7500 Fast real-time PCR system: initial denaturation at 95°C for 10 min, followed by 40 cycles of denaturation at 95°C for 15 s, annealing at 59 to 60°C for 30 s, and elongation at 72°C for 30 s. Following amplification, a melting curve analysis was used to assess the contribution of primer-dimer formation or nonspecific target amplification to the desired product signal. qPCR primers used in this work are detailed in Table 1. Amplicons were quantified using the ΔΔ*C_T_* method (where *C_T_* is threshold cycle) of relative gene expression analysis, expressed as fold changes (44). Comparisons of gene expression levels in the text between two stimulation conditions are listed as mean fold changes between the two conditions. Secretion data between two conditions are reported as the fold change (in picograms per milliliter) between the two conditions.

**TABLE 1:** Primers used for qRT-PCR analysis

Target gene symbol	GenBank accession no. of target	Primer (direction)	Sequence (5′ → 3′)
*IL23A*	XM_011538477.2	*IL23* (forward)	AAC TGA GGG AAC CAA ACC AG
*IL23A*	XM_011538477.2	*IL23* (reverse)	ATC TCT GCC CAC TCC CAC TT
*IL6*	XM_005249745.5	*IL6* (forward)	CCA CTC ACC TCT TCA GAA CG
*IL6*	XM_005249745.5	*IL6* (reverse)	CAT CTT TGG AAG GTT CAG GTT G
*IL1β*	XM_017003988.2	*IL1β* (forward)	TCT ACA CCA ATG CCC AAC TG
*IL1β*	XM_017003988.2	*IL1β* (reverse)	AAG TGA GTA GGA GAG GTG AGA G
Cathepsin B	NM_001317237.1	Cathepsin B (forward)	CTG GCT GTA ATG GTG GCT ATC
Cathepsin B	NM_001317237.1	Cathepsin B (reverse)	GCA CCC TAC ATG GGA TTC ATA G

### Cytokine secretion measurement.

The supernatant recovered from THP-1 stimulation experiments was used to measure the secreted cytokines IL-1β, IL-6, and IL-23. Cytokines were measured using the human IL-23 DuoSet, human IL-6 DuoSet, and human IL-1β DuoSet enzyme-linked immunosorbent assay (ELISA) kits according to the manufacturer’s instructions (R&D Systems, Minneapolis, MN, USA).

### Statistical analysis.

In all the experimental replicates included in this analysis, stimulation with live V. cholerae generated the highest IL-23 response, and therefore, we considered this condition to be a positive control for stimulation (mean value, 4,861 pg/ml; range, 1,079 to 8,871 pg/ml). To avoid including experiments in which cells were not adequately stimulated, we excluded replicates without >1,000 pg/ml of IL-23 secretion in cells stimulated with live wild-type V. cholerae. Medium alone (with no organisms or toxin added) was considered a negative control. The average medium value was 91 pg/ml (range, 39 to 302 pg/ml). Experimental values were normalized by medium measurements from the same experiment for each replicate. We also examined variation within the positive (live V. cholerae) and negative (medium alone) controls for each experimental run by calculating the standard deviation of the values for the positive-control wells divided by the standard deviation of the values for the negative-control wells, and this number was divided by the absolute value of the mean of positive-control values minus the mean of the negative-control values (modified Z factor) and excluded experiments with a score of less than 0.6, indicating a high level of well-to-well variation in the control replicates relative to the overall signal window between the positive and negative controls (45). Comparisons of levels of cytokine secretion and expression were made by nonparametric paired Wilcoxon testing comparing means, and for unpaired data, nonparametric *t* testing by the Mann-Whitney rank test was used. For paired, parametric testing for small sample sizes, the paired *t* test was used. *P* values of <0.05 were considered statistically significant. We performed the analyses and generated the figures using GraphPad Prism 7 (GraphPad Software, Inc., La Jolla, CA, USA).

### Data availability.

Full data on the RNA expression measured under each of the conditions (live V. cholerae, inactivated V. cholerae, CT, and CTB) are available under accession number PRJNA529418 in the NCBI Sequence Read Archive.
