# Supplementary figures and images for: Posttranslational Regulation of IL-23 Production Distinguishes the Innate Immune Responses to Live Toxigenic versus Heat-Inactivated Vibrio cholerae
Source: mSphere. 2019 Aug 21;4(4):e00206-19. doi: 10.1128/mSphere.00206-19 (PMC6706466; doi:10.1128/mSphere.00206-19)

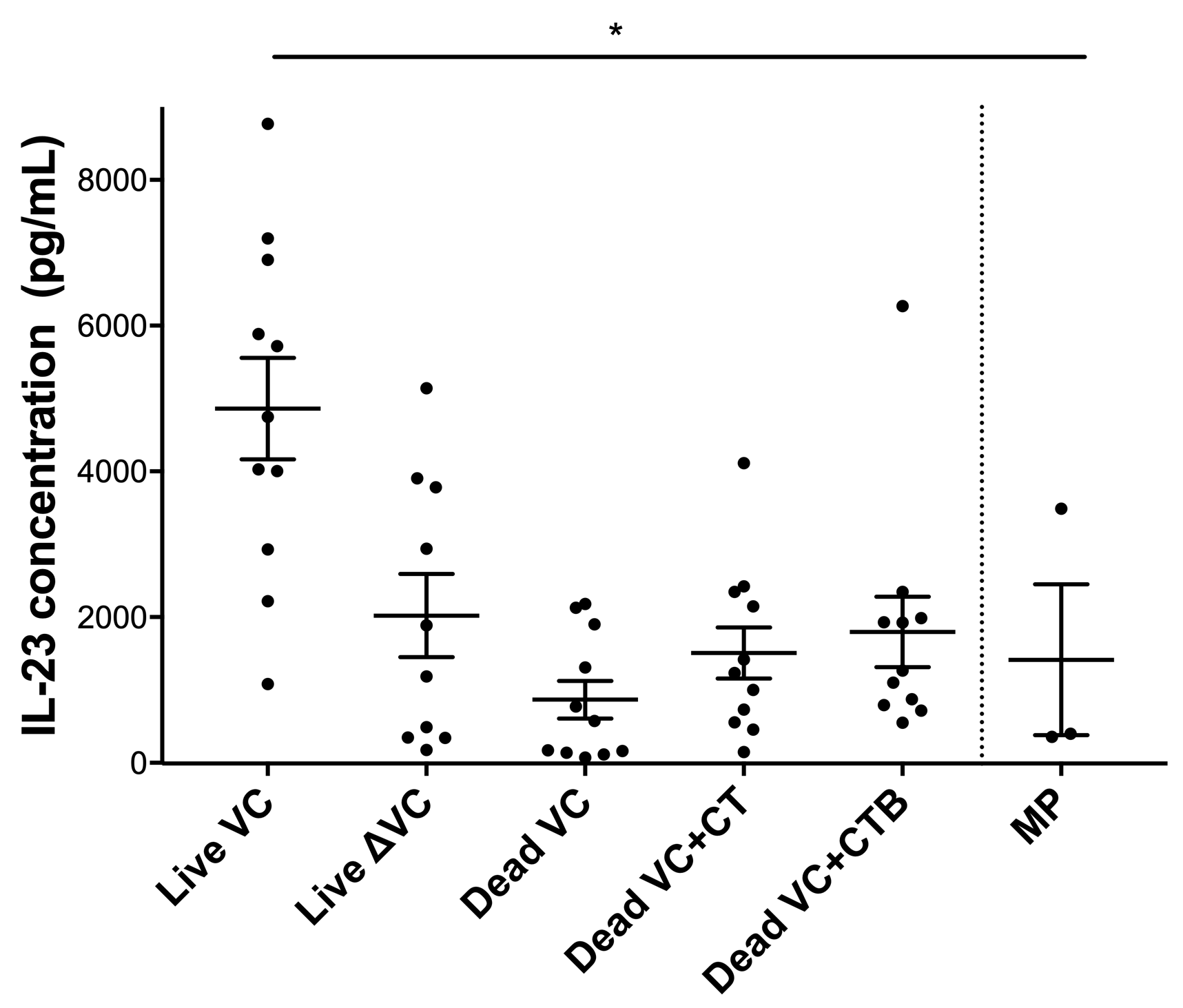

Supplement: FIG S1 [file mSphere.00206-19-sf001.tif]
